# Rebamipide prevents peripheral arthritis and intestinal inflammation by reciprocally regulating Th17/Treg cell imbalance in mice with curdlan-induced spondyloarthritis

**DOI:** 10.1186/s12967-016-0942-5

**Published:** 2016-06-27

**Authors:** Hong-Ki Min, Jae-Kyung Kim, Seon-Yeong Lee, Eun-Kyung Kim, Seung Hoon Lee, Jennifer Lee, Seung-Ki Kwok, Mi-La Cho, Sung-Hwan Park

**Affiliations:** Division of Rheumatology, Department of Internal Medicine, College of Medicine, The Catholic University of Korea, Seoul, 137-701 South Korea; Rheumatism Research Center, Catholic Research Institute of Medical Science, The Catholic University of Korea, Seoul, 137-040 South Korea

**Keywords:** Spondyloarthritis, Rebamipide, Type 17 helper T cell, Regulatory T cell

## Abstract

**Background:**

Spondyloarthritis (SpA) usually manifests as arthritis of the axial and peripheral joints but can also result in extra-articular manifestations such as inflammatory bowel disease. Proinflammatory cytokine interleukin-17 (IL-17) plays a crucial role in the pathogenesis of SpA. Rebamipide inhibits signal transducer and activator of transcription 3 that controls IL-17 production and Th17 cell differentiation. This study examined the effect of rebamipide on SpA development.

**Methods:**

SKG ZAP-70^W163C^ mice were immunized with curdlan to induce SpA features. The mice were then intraperitoneally injected with rebamipide or vehicle 3 times a week for 14 weeks and their clinical scores were evaluated. Histological scores of the paw and spine and the length of the gut were measured at sacrifice. Immunohistochemical staining of IL-17 and tumor necrosis factor-α (TNF-α) was performed using tissue samples isolated from the axial joints, peripheral joints, and gut. Spleen tissue samples were isolated from both rebamipide- or vehicle-treated mice with SpA at 14 weeks after curdlan injection to determine the effect of rebamipide on Th17 and regulatory T (Treg) cell differentiation.

**Results:**

Rebamipide decreased the clinical and histological scores of the peripheral joints. The total length of the gut was preserved in rebamipide-treated mice. IL-17 and TNF-α expression in the spine, peripheral joints, and gut was lower in rebamipide-treated mice than in control mice. Th17 cell differentiation was suppressed whereas Treg cell differentiation was upregulated in the spleen of rebamipide-treated mice.

**Conclusion:**

Rebamipide exerted beneficial effects in mice with SpA by preventing peripheral arthritis and intestinal inflammation and by regulating Th17/Treg cell imbalance, suggesting that it can be used as a potential therapeutic agent for treating arthritis to SpA patients.

## Background

Spondyloarthritis (SpA) is a chronic immune-mediated inflammatory arthritis that usually accompanies sacroiliitis and spondylodiscitis. SpA can cause inflammation of the peripheral joints and extra-articular organs such as the intestine, eye, and skin. SpA can be classified into ankylosing spondylitis (AS), psoriatic arthritis (PsA), reactive arthritis, arthritis associated with inflammatory bowel disease, and undifferentiated SpA based on its clinical manifestations [1].

Recent studies have shown that interleukin-23 (IL-23) and IL-17 play a crucial role in the pathogenesis of SpA [2, 3]. IL-23 is mainly secreted by dendritic cells and macrophages and acts on various innate and adaptive immune cells to release cytokines, including IL-17, TNF-α, and IL-22, in target tissues, thus leading to synovitis, enthesitis, gut inflammation, and osteoproliferation [2]. IL-17 levels are elevated in the serum and synovial fluids of patients with SpA [4, 5]. In addition, patients with SpA show increased number of type 17 helper T (Th17) cells in the peripheral blood [6, 7]. A study on a mouse model of SpA indicated that IL-17 was involved in the development of enthesitis and ileitis [8]. Recent clinical trials on the treatment of AS and PsA with anti-IL-17 monoclonal antibodies (Abs) have provided promising results [9, 10].

Th17 and regulatory T (Treg) cells exert opposite effects on the pathogenesis of autoimmune diseases. An imbalance in the number of Th17 and Treg cells (increased number of Th17 cells and decreased number of Treg cells) is suggested to be associated with the pathogenesis of SpA [6, 11–13]. Xueyi et al. [14] suggested that TNF-α-blocking agents exerted beneficial effects in patients with AS by suppressing TNF-α and by regulating Th17/Treg cell imbalance. These findings support the hypothesis that Th17/Treg cell imbalance is involved in the pathogenesis of SpA.

Rebamipide is widely used as a gastroprotective agent. In addition, it exerts antioxidant effects and inhibits signal transducer and activator of transcription 3 (STAT3) [15]. Rebamipide exerted beneficial effects in a mouse model of rheumatoid arthritis (RA) by regulating Th17/Treg cell imbalance by modulating *STAT3* and *FoxP3* expression [15].

ZAP-70^W163C^ mutation impairs T cell receptor signaling and induces the production of highly autoreactive T cells. Immunization of SKG ZAP-70^W163C^ mice with 1,3-β-glucan (curdlan) triggers the development of SpA features by activating dectin 1/Syk signaling pathway [16]. A study showed that SpA features could be transferred by CD4^+^ cells isolated from curdlan-immunized SKG ZAP-70^W163C^ mice, indicating that adaptive immunity played a crucial role in the pathogenesis of SpA [16]. Therefore, the aforementioned mouse model of SpA was found to be suitable for examining the effects of rebamipide on CD4^+^ T cells.

The present study determined whether rebamipide exerted beneficial effects in a mouse model of SpA. For this, we measured clinical score, histological score, length of the gut, and expression of cytokines in affected tissues. In addition, we determined the effects of rebamipide on CD4^+^ T cells by assessing Th17 and Treg cell populations in splenocytes isolated from rebamipide- and vehicle-treated mice with SpA.

## Methods

### Mice

SKG mice with BALB/c background were kindly donated by Professor Shimon Sakaguchi (Department of Experimental Immunology, World Premier International Immunology Frontier Research Center, Osaka University). These mice were maintained under specific pathogen-free (SPF) condition and were fed standard mouse chow (Ralston Purina, St Louis, MO, USA) and water ad libitum. All experimental procedures were assessed and approved by the Institutional Animal Care and Use Committee of the School of Medicine and the Animal Research Ethics Committee of the Catholic University of Korea (CUMC-2015-0063-01) and were conducted in accordance with the Laboratory Animals Welfare Act, Guide for the Care and Use of Laboratory Animals.

### SpA induction and rebamipide treatment

Curdlan (3 mg/kg) was injected intraperitoneally (i.p) into SKG mice aged 8–10 weeks. The mice were then i.p injected with rebamipide (6 mg/kg) or vehicle 3 times a week for 14 weeks. Clinical scores were monitored weekly for prior 7 weeks and twice a week for later 7 weeks: 0 = no swelling or redness, 0.1 = swelling or redness of the digits, 0.5 = mild swelling and/or redness of the wrist or ankle joints, and 1 = severe swelling of larger joints [16]. Scores of affected joints were summed for each mouse.

### Histopathological analysis

Formalin-fixed tissue samples from the peripheral joints, spine, colon, and small intestine were embedded in paraffin, and 7-µm sections were prepared. The sections were dewaxed using xylene, dehydrated using an alcohol gradient, and stained with hematoxylin and eosin (H&E). The H&E-stained sections of the peripheral joints and spine were scored for inflammation. Histological score of the peripheral joints was determined on a scale of 1–4: 1 = few infiltrating immune cells, 2 = 1–2 small patches of inflammation, 3 = inflammation throughout the ankle joint, and 4 = inflammation in the soft tissue (enthesitis or fasciitis). Histological score of the spine was determined on a scale of 1–4: 1 = few infiltrating immune cells, 2 = mild inflammation of the discs or along the vertebrae (0–30 % of the discs), 3 = inflammation of the discs and/or along the vertebrae (30–70 % of the discs), and 4 = inflammation of 70 % of the discs and along the vertebrae [16].

### Immunohistochemistry

Immunohistochemical analysis was performed using Vectastain ABC Kit (Vector Laboratories, Burlingame, CA, USA). The tissues were first incubated with primary Abs against IL-17 and TNF-α (Santa Cruz Biotechnology, Santa Cruz, CA, USA) overnight at 4 °C and then with biotinylated secondary Abs against goat (Santa Cruz Biotechnology) and streptavidin–peroxidase complex (Vector Laboratories, Burlingame, CA, USA) for 1 h. The final colored product was developed using a chromogen 3,3-diaminobenzidine (Dako, Carpinteria, CA, USA). All histological assessments were performed by two independent, blinded observers. Images were captured using DP71 digital camera (Olympus, Center Valley, PA, USA) attached to BX41 microscope (Olympus) at 3400 magnification.

### Confocal microscopy

Spleen tissues were isolated from mice in both the group at 14 weeks after curdlan injection. To examine the populations of Th17 and Treg cells, the tissues were stained with Abs against CD4–fluorescein isothiocyanate (FITC), IL-17–phycoerythrin (PE), CD25–allophycocyanin, and FOXP3–PE (all from eBioscience, San Diego, CA, USA). To determine the population of cells expressing STAT, the tissues were stained with Abs against CD4–FITC, *p*-STAT3–Y705–PE, and *p*-STAT3–S727–PE (eBioscience, San Diego, CA, USA). The stained tissue sections were analyzed using a confocal microscope (LSM 510 Meta; Carl Zeiss, Oberkochen, Germany). We counted cell number using Pannoramic MIDI and Pannoramic viewer (3D HISTECH Ltd, Hungary).

### Flow cytometric analyses

Cell pellets were prepared from the spleen tissues isolated from rebamipide- and vehicle-treated mice with SpA. Populations of T cells were examined by staining the tissues with a monoclonal Ab against CD4–peridin chlorophyll protein (eBioscience). Cells were permeabilized and fixed with CytoFix (BD Biosciences, San Jose, CA, USA), as instructed by the manufacturer, and were stained with Abs against IL-17–PE and IL-10–FITC (eBioscience).

### Statistical analysis

All data are expressed as mean ± SEM. Statistical analysis was performed using SPSS 20.0 for Windows (IBM Corp., Armonk, NY, USA). Differences between the two groups were analyzed using Mann–Whitney test by assuming equal variances. *P* < 0.05 was considered significant.

## Results

### Rebamipide prevents peripheral arthritis in mice with SpA

**Fig. 1:**
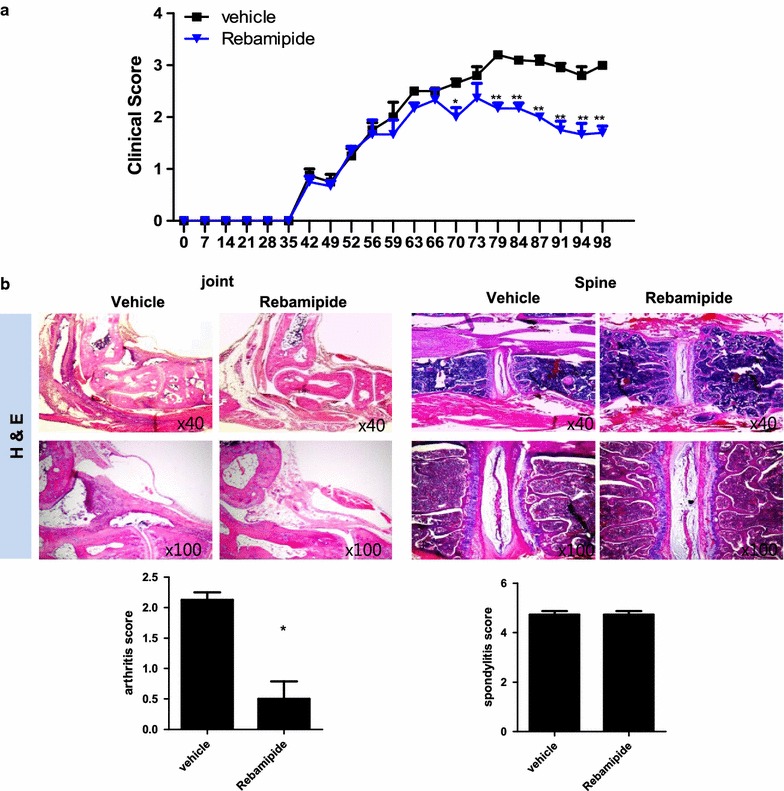
Rebamipide prevents arthritis in mice with SpA. Mice were i.p. injected with rebamipide 3 times a week after SpA induction. **a** Mean clinical score was determined based on arthritis severity. **b** Joint and spine tissues were isolated from rebamipide-treated and vehicle-treated mice with SpA at 14 weeks after curdlan injection and were stained with H&E. Inflammation and cartilage scores are shown in the *bar graphs*. Data are represented as the mean ± SEM of 3 independent experiments (*P < 0.05, **P < 0.005)

We investigated whether rebamipide prevented peripheral arthritis in the mouse model of SpA. Mice with SpA were i.p injected with rebamipide (6 mg/kg) or vehicle 3 times a week after curdlan injection. The mice were sacrificed at 14 weeks after curdlan injection and their joint tissues were isolated (n = 5 for each group). Clinical scores of rebamipide-treated mice were significantly lower than those of vehicle-treated mice (Fig. 1a, 1.8 ± 0.12 vs. 3.0 ± 0.06, *P* = 0.007). Arthritis scores (histological scores of the peripheral joints) were significantly decreased in rebamipide-treated mice (Fig. 1b, 0.7 ± 0.19 vs. 2.2 ± 0.10, *P* = 0.035). However, spondylitis scores (histological scores of the spine) were not significantly different between rebamipide- and vehicle-treated mice (Fig. 1b). These results indicated that rebamipide exerted a substantial preventive effect on peripheral arthritis in mice with SpA.

### Rebamipide suppresses proinflammatory cytokine expression in the peripheral joints and spine of mice with SpA

**Fig. 2:**
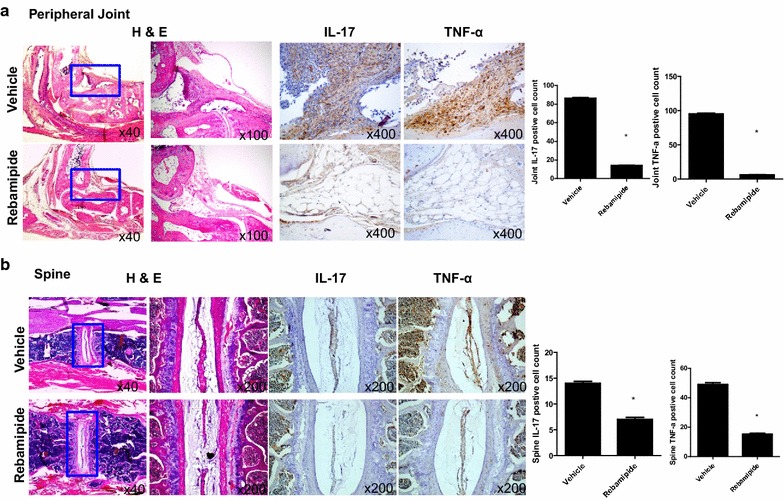
Rebamipide suppresses proinflammatory cytokine expression in the affected peripheral joints of mice with SpA. **a** and **b** Peripheral joint and spine tissues were stained with H&E and then with specific Abs against IL-17 and TNF-α. IL-17- or TNF-α-positive cells are shown in the *bar graphs* (*right*). Data are represented as the mean ± SEM of 3 independent experiments (*P < 0.05)

Peripheral joint and spine tissues (n = 5 for each group) isolated from rebamipide- and vehicle-treated mice were stained with Abs against IL-17 and TNF-α. IL-17 and TNF-α expression was suppressed in the peripheral joints of rebamipide-treated mice (Fig. 2a, 14.0 ± 0.30 vs. 86.0 ± 0.64 %, *P* = 0.029 and 6.0 ± 0.28 vs. 95.0 ± 1.00 %, *P* = 0.029). Moreover, IL-17- and TNF-α-expressing cells significantly decreased in the spine tissues of rebamipide-treated mice; however, this difference was lower than that observed in the peripheral joint tissues (Fig. 2b, 7.0 ± 0.38 vs. 14.0 ± 0.32 %, *P* = 0.029 and 15.3 ± 0.51 vs. 49.0 ± 1.00 %, *P* = 0.029). These findings were consistent with the aforementioned histological scores, suggesting that rebamipide more substantially suppressed proinflammatory cytokine expression in the peripheral joints than in the axial joints.

### Rebamipide prevents gut inflammation in mice with SpA by suppressing IL-17 and TNF-α expression in the gut

**Fig. 3:**
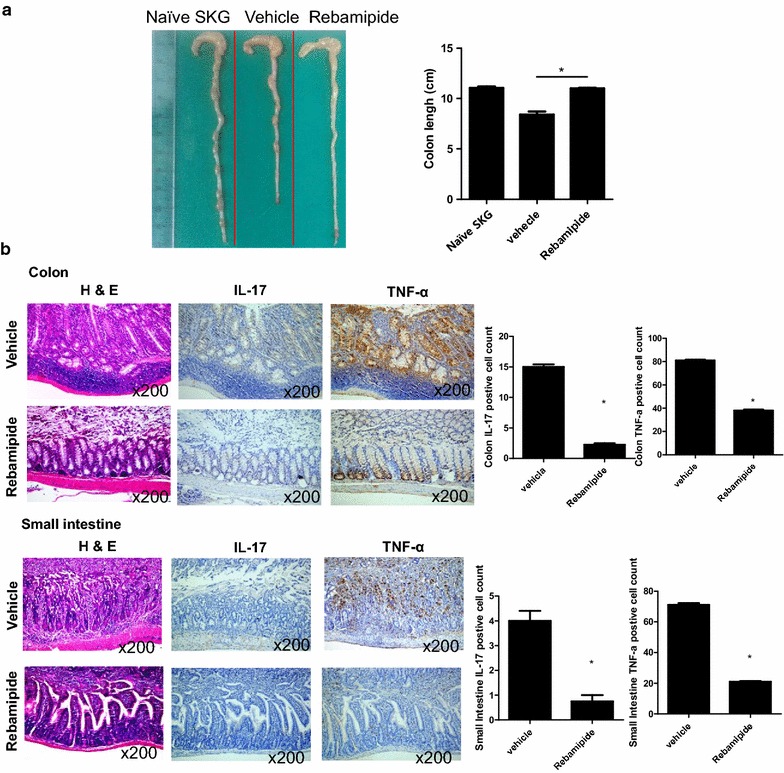
Rebamipide prevents gut inflammation in mice with SpA by suppressing IL-17 and TNF-α expression in the gut. **a** Gut length is shown in the *bar graph*. **b** Colon and small intestine tissues were stained with H&E and then with specific Abs against IL-17 and TNF-α. IL-17- or TNF-α-positive cells are shown in the *bar graph* (*right*). Data are represented as the mean ± SEM of 3 independent experiments (*P < 0.05)

Gut tissues were isolated in the same manner as joint tissues (n = 5 for each group). The total length of the gut of rebamipide-treated mice with SpA was the same as that of naive SKG mice. However, the total length of the gut of vehicle-treated mice with SpA was distinctly shortened (Fig. 3a, 8.5 ± 0.23 vs. 11.0 ± 0.04 cm, *P* = 0.035). IL-17 and TNF-α expression significantly decreased in tissues isolated from both the large and small intestines of rebamipide-treated mice (Fig. 3b, 2.0 vs. 15.0 ± 0.36 %, *P* = 0.019 and 38.0 ± 0.62 vs. 81.0 ± 0.64 %, *P* = 0.029 and 1.0 vs. 4.0 ± 0.28 %, *P* = 0.029 and 21.0 ± 0.24 vs. 71.0 ± 1.00 %, *P* = 0.029). These results indicated that rebamipide exerted gastroprotective effects in mice with SpA by regulating IL-17 and TNF-α expression.

### Rebamipide reciprocally regulates Th17/Treg cell imbalance

**Fig. 4:**
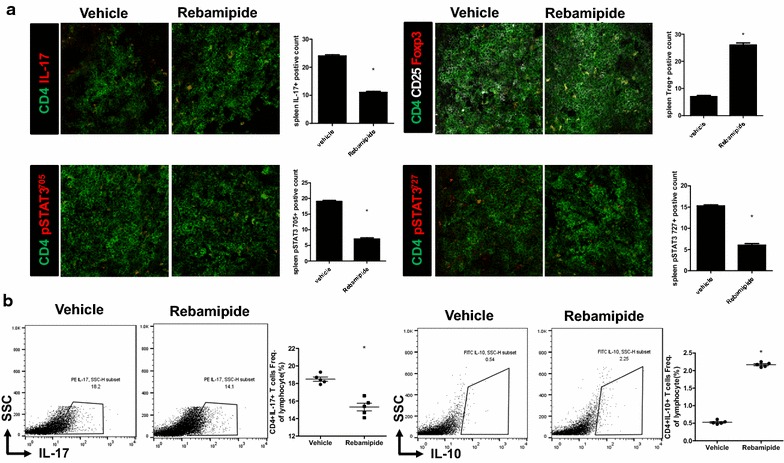
Rebamipide reciprocally regulates Th17/Treg cell imbalance. Spleen tissues were isolated from mice in each group at 14 weeks after curdlan injection. **a** The tissues were stained with specific Abs against CD4 (*green*), CD25 (*white*), IL-17 (*red*), FOXP3 (*green*), *p*-STAT3–Y705 (*red*), and *p*-STAT3–S727 (*red*). Positive cells are shown in the *bar graphs*. **b** Spleen tissues were isolated from mice in each group at 14 weeks after curdlan injection. Splenocytes were stained with Abs against CD4–PerCP, IL-17–PE, and IL-10–FITC to determine the presence of Th17 and IL-10-expressing Treg cells. Data are expressed as the mean ± SEM of 3 independent experiments (*P < 0.05)

Spleen tissues were isolated from vehicle- and rebamipide-treated mice with SpA (n = 5 for each group). The number of CD4^+^*p*-STAT3(Y705)^+^, CD4^+^*p*-STAT3(S727)^+^, and CD4^+^IL-17^+^ cells was significantly lower in spleen tissues isolated from rebamipide-treated mice than in those isolated from vehicle-treated mice (Fig. 4a, 7.0 ± 0.40 vs. 19.0 ± 0.36 %, *P* = 0.029 and 6.0 ± 0.32 vs. 15.3 ± 0.19 %, *P* = 0.026 and 11.0 ± 0.34 vs. 24.0 ± 0.24 %, *P* = 0.029). The number of CD4^+^CD25^+^FOXP3^+^ cells was significantly higher in spleen tissues isolated from rebamipide-treated mice (Fig. 4a, 26.0 ± 0.64 vs. 7.0 ± 0.32 %, *P* = 0.029). Flow cytometric analysis of splenocytes isolated from mice in each group showed that the number of Th17 cells significantly decreased while that of IL-10-expressing Treg cells increased in rebamipide-treated mice (Fig. 4b, 15.3 ± 0.35 vs. 18.5 ± 0.19 %, *P* = 0.028 and 2.2 ± 0.02 vs. 0.5 ± 0.02 %, *P* = 0.028). These results are consistent with those of the study that showed rebamipide reciprocally regulated Th17/Treg cell imbalance in the mouse model of RA [15].

## Discussion

The present study showed that rebamipide exerted preventive effects on peripheral arthritis and gut inflammation in mice with SpA. Gross appearance and histology showed the definite antiarthritic effect of rebamipide on the peripheral joints of mice with SpA. In addition, rebamipide preserved the length of the gut and decreased the expression of proinflammatory cytokines in the gut tissue, indicating that it exerted beneficial effects on gut inflammation in mice with SpA. These beneficial effects could be explained by 2 mechanisms: (1) suppression of IL-17 and TNF-α expression in the peripheral joints and gut tissues and (2) regulation of Th17/Treg cell imbalance.

The IL-23/IL-17 axis plays a critical role in the pathogenesis of SpA [2, 3]. Antigen-presenting cells (APCs) such as dendritic cells and macrophages are a major source of IL-23 [2]. IL-23 induces immune responses by affecting both innate and adaptive immune cells. Various immune cells, including Th17 cells, are involved in downstream immune reactions that contribute to the pathogenesis of SpA [2]. However, limited information is available on the role of each immune cell type in the pathogenesis of SpA. Dominant IL-17-expressing cells differ depending on their location, such as myeloperoxidase-positive neutrophils in the facet joints and c-Kit-positive mast cells in the synovium [17, 18]. Some studies have suggested that innate immune response plays a dominant role in the pathogenesis of SpA while some studies have indicated that helper T cells play a dominant in the pathogenesis of SpA. A study on mice with curdlan-induced SpA indicated that SpA features could be transferred to mice with severe combined immunodeficiency by using CD4^+^ T cells [16]. Non-responders to anti-TNF-α therapy showed persistently high number of Th17 cells and low number of Treg cells while responders showed a regulation of Th17/Treg cell imbalance [14]. Moreover, the number of Th17 cells in these patients was positively correlated with Bath ankylosing spondylitis disease activity index (BASDAI) and Bath ankylosing spondylitis functional index (BASFI), whereas the number of Treg cells was negatively correlated with BASDAI and BASFI [14]. The main pathologic cells involved in the pathogenesis of SpA are still unknown; however, it is certain that CD4^+^ T cells are involved in the pathogenesis of SpA. The present study showed that rebamipide reciprocally regulated Th17/Treg cell imbalance in mice with SpA. However, rebamipide only exerted beneficial effects on the peripheral joints and gut and did not prevent inflammation in the axial joints. These findings suggest that only Th17/Treg cell imbalance is associated with peripheral arthritis and gut inflammation. However, further studies should be performed to elucidate the mechanism underlying the systemic effect of Th17/Treg cell imbalance on tissue-specific pathologic cells involved in the pathogenesis of SpA.

We observed that IL-17 and TNF-α expression decreased in the peripheral joints and gut (where rebamipide exerted beneficial effects) and in the axial joints. These cytokines play a major role in the development of synovitis and gut inflammation [2]. The degree of decrease in IL-17 and TNF-α expression in the spine was lower than that in the peripheral joints, which may be the reason why rebamipide did not exert protective effects in the spine. Although rebamipide did not show beneficial effect on spondylitis, rebamipide is relatively safe drug than conventional drugs including nonsteroidal anti-inflammatory drug (NSAID), immunosuppressants, and TNF-α blocker [19]. Furthermore, rebamipide could ameliorate NSAID-induced gastropathy [19, 20]. Therefore, rebamipide could be added to SpA patients without critical side effect while maintaining current medications.

STAT3 signaling regulates the differentiation of CD4^+^ T cells into Th17 cells [21, 22]. Rebamipide inhibited STAT3 and regulated Th17/Treg cell imbalance in the mouse model of RA [15]. In another study, rebamipide exerted similar effects on Th17/Treg cell imbalance in a mouse model of RA [23]. The present study showed that rebamipide regulated Th17/Treg cell imbalance in mice with curdlan-induced SpA by modulating the activation of STAT3 and FOXP3. However, future studies should be performed to evaluate the effect of rebamipide on new bone formation because rebamipide acts as a STAT3 inhibitor. In a mouse model of collagen antibody-induced SpA, STAT3 activation was associated with osteoblast-mediated new bone formation [24]. A study involving mice with curdlan-induced SpA used micro-CT scan to show that curdlan injection induced new bone formation at 12 weeks. However, this study did not show definite syndesmophytes on histological findings [16]. In the present study, vehicle-treated mice with SpA did not show new bone formation on histological findings. This may be because of an insufficient follow-up period. Therefore, future studies involving longer follow-up periods should be performed to verify the effects of rebamipide on new bone formation in mice with SpA.

## Conclusions

In conclusion, rebamipide exerted beneficial effects in the mouse model of curdlan-induced SpA. Rebamipide prevented the development of peripheral arthritis and gut inflammation and suppressed the expression of major pathologic cytokines such as IL-17 in the peripheral joints, spine, and gut. Further, rebamipide reciprocally regulated Th17/Treg cell imbalance. These results suggest that rebamipide could be used as a therapeutic agent for treating arthritis to SpA patients.

## Abbreviations

Abs: antibodies
APCs: antigen-presenting cells
AS: ankylosing spondylitis
BASDAI: bath ankylosing spondylitis disease activity index
BASFI: bath ankylosing spondylitis functional index
FITC: fluorescein isothiocyanate
FOXP3: forkhead box P3
H&E: hematoxylin and eosin
IL: interleukin
i.p: intraperitoneal
NSAID: nonsteroidal anti-inflammatory drug
PsA: psoriatic arthritis
PE: phycoerythrin
RA: rheumatoid arthritis
SpA: spondyloarthritis
SPF: under specific pathogen-free
STAT3: signal transducer and activator of transcription 3
Th: helper T cell
TNF-α: tumor necrosis factor-α
Treg: regulatory T cell
